# Teaching Literacy Skills to French Minimally Verbal School-Aged Children with Autism Spectrum Disorders with the Serious Game SEMA-TIC: An Exploratory Study

**DOI:** 10.3389/fpsyg.2017.01523

**Published:** 2017-09-05

**Authors:** Sylvie Serret, Stéphanie Hun, Susanne Thümmler, Prescillia Pierron, Andreia Santos, Jérémy Bourgeois, Florence Askenazy

**Affiliations:** ^1^Autism Resources Center, Lenval Foundation, Child and Adolescent Psychiatry Department, Children's Hospitals of Nice CHU-Lenval Nice, France; ^2^EA 7276 CoBTeK – Cognition Behaviour Technology, University of Nice Sophia-Antipolis, Claude Pompidou Institute, Edmond and Lily Safra Center Nice, France; ^3^Child and Adolescent Psychiatry Department, Children's Hospitals of Nice CHU-Lenval Nice, France

**Keywords:** autism spectrum disorders, reading skills, literacy skills, serious games, non-verbal cognition

## Abstract

Learning to read is very challenging for children with Autism Spectrum Disorders (ASD), but also very important, as it can give them access to new knowledge. This is even more challenging in minimally verbal children, who do not have the verbal abilities to learn through usual methods. To address the learning of literacy skills in French minimally verbal school-aged children with ASD, we designed the serious game SEMA-TIC, which relies on non-verbal cognitive skills and uses specific learning strategies adapted to the features of autistic individuals. This study investigated the usability of SEMA-TIC (in terms of adaptability, efficiency, and effectiveness) for the acquisition of literacy skills in French minimally verbal school-aged children with ASD. Twenty-five children with ASD and no functional language participated in the study. Children in the training group received the SEMA-TIC training over 23 weeks (on average), while no intervention was provided to children in the non-training group. Results indicated that SEMA-TIC presents a suitable usability, as all participants were able to play (adaptability), to complete the training (efficiency) and to acquire significant literacy skills (effectiveness). Indeed, the literacy skills in the training group significantly improved after the training, as measured by specific experimental tasks (alphabet knowledge, word reading, word-non-word discrimination, sentence reading and word segmentation; all *p* ≤ 0.001) compared to the non-training group. More importantly, 3 out of 12 children of the training group could be considered as word decoders at the end of the intervention, whereas no children of the non-training group became able to decode words efficiently. The present study thus brings preliminary evidence that French minimally verbal school-aged children with ASD are able to learn literacy skills through SEMA-TIC, a specific computerized intervention consisting in a serious game based on non-verbal cognitive skills.

## Introduction

Autism Spectrum Disorders (ASD) are complex and heterogeneous disorders, characterized by deficient social communication and by repetitive and stereotyped behaviors (American Psychiatric Association, 2013). In addition to these core features, intellectual and language levels are the two main prognostic factors for the severity of autism at adult age (Howlin et al., 2004).

It is widely recognized that about 30% of children with ASD remain minimally verbal after age 6, even after receiving years of behavioral, developmental and educational interventions (Anderson et al., 2007). Although there is a lack of consensus, minimally verbal school-aged children are characterized by the use of a very limited repertoire (less than 20 functional words) of spoken, non-echoed or scripted language for the purpose of communication (Kasari et al., 2013). Because of their poor verbal communication, some of these children are sometimes considered as “low-functioning,” due to difficulties in performing classical assessments of global cognitive skills. However, minimally verbal children with ASD are not necessarily characterized by severe levels of intellectual disability (Tager-Flusberg and Kasari, 2013).

Due to the peculiarities of ASD, many children experience significant difficulties in learning to read (Ramdoss et al., 2012), and the access to new knowledge can thus be challenging. Indeed, reading is traditionally regarded as “the most important academic skill learned in school” (Mastropieri and Scruggs, 1997; Nation et al., 2006) because children “learn to read” so that they can “read to learn.”

In children with typical development, it is assumed that spoken language emerges before the ability to read, thus making efficient oral language one of the most important predictive factors for successful reading skills (Miranda, 2003; Muter et al., 2004; Clarke et al., 2010; Nation et al., 2010; St Clair et al., 2011).

The Simple View of Reading proposes that reading comprehension implies both word recognition and oral language comprehension (Hoover and Gough, 1990). These literacy skills may indeed include phonological awareness (i.e., the ability to identify and manipulate sounds in speech), phonics (i.e., the knowledge of letter–sound correspondences and their use in reading and spelling, decoding unfamiliar words), fluency (i.e., reading with speed, accuracy, and proper expression), vocabulary (i.e., the understanding of word meanings), and comprehension (i.e., the construction of meaning from text). Other important factors for successful reading consist of non-verbal cognitive abilities (Howlin et al., 2004; Lidstone et al., 2009), literacy basic knowledge, and motivation (Becker et al., 2010). In line with these ideas, children with typical development are classically taught to read by decoding words through the recognition and the manipulation of the correspondence between letters and sounds (Randi et al., 2010).

However, it appears that the learning to read is complex in children with ASD, with large discrepancies among individuals concerning their reading skills (Nation et al., 2006; Newman et al., 2007; Jones et al., 2009; Lindgren et al., 2009). While their decoding skills are often described as correct, although heterogenous (Nation et al., 2006), reading comprehension skills are usually poor in individuals with ASD (Jones et al., 2009; Huemer and Mann, 2010; Ricketts et al., 2013). Letter-sound correspondence and phonemic awareness are indeed considered as critical skills in children at risk of reading difficulties (Benedek-Wood et al., 2015).

The learning of reading in minimally verbal children with ASD is even more complex, because of their lack of oral language skills. Consequently, very few of these children succeed in developing literacy skills beyond sight word recognition (Vacca, 2007). Indeed, very little research and learning interventions do exist in this population (Mucchetti, 2013), even though literacy skill acquisition might be a support for minimally verbal children with ASD to access opportunities for greater academic performance, and may contribute to improve their communication (Benedek-Wood et al., 2015). Interestingly, it was found that some individuals with ASD were able to read and write meaningfully despite the absence of spoken language (Goh et al., 2013).

Indeed, minimally verbal children with ASD have cognitive potential other than verbal-based skills (Munson et al., 2008; Courchesne et al., 2015), including efficient spatial cognition (Edgin and Pennington, 2005) and good memory for material with low levels of structure (Williams et al., 2006). Analogical reasoning (Morsanyi and Holyoak, 2010) and implicit learning (Barnes et al., 2008; Brown et al., 2010; Nemeth et al., 2010) even appear as cognitive peaks in people with ASD, independently of the overall IQ.

Previous studies support the idea of an advantage for visual-graphical than for phonological processes in individuals with ASD (Kamio and Toichi, 2000; Koshino et al., 2005). In addition, individuals with ASD rely more on visual imagery to understand sentences than individuals without ASD, suggesting that they may employ visual thinking to compensate for language limitations (Kana et al., 2006). It has also been suggested that the functioning of neural networks that underlie the visual decoding of words may be enhanced in individuals with ASD (Grigorenko et al., 2003). In line with these studies, recent evidence suggests that not only oral language performance, but also non-verbal cognition plays a significant role in meaning-related emergent literacy skills in children with ASD (Westerveld et al., 2017). These findings may provide the basis to develop new ways of teaching academic knowledge—such as literacy skills—in minimally verbal children with ASD relying mainly on their non-verbal abilities and not on phonological processes. Recent animal studies suggested that literacy skills can be learned without the implication of any phonological abilities. Grainger et al. (2012) demonstrated that baboons can learn the statistics of letter co-occurrence inherent in the English language, so that they become able to distinguish between written words and non-words. The Visual Word Form Area in the left occipito-temporal sulcus region is selectively activated when reading written characters, and not when recognizing spoken words.

Previous literature reviews underline the need of specialized reading instruction interventions in order to improve the reading abilities of children with ASD, targeting both code-focused and meaning-focused reading instructions (Whalon et al., 2009; Tager-Flusberg and Kasari, 2013). In contrast, the interest of sight word reading approach for children with ASD with cognitive and verbal limitations should not be neglected, as sight word instruction allow the identification of printed words (Spector, 2011). Indeed, while identifying sounds in words, mapping them to corresponding letters and combining sounds together to form words should be possible regardless of the IQ of children with ASD (Tjus et al., 1998; Browder et al., 2006), it appears much more complex for children with ASD with verbal limitations (Whalon et al., 2009). Alternative approaches, not relying on phonological processes, appear thus necessary in this specific population. However so far, most literacy instructions have focused on isolated skills, in decontextualized, behavioral approaches (Katims, 2000). Moreover, only few studies addressed basic literacy and skills in minimally verbal children with ASD, while a recent study focusing on teaching key pre-verbal skills suggested that teaching reading and writing skills might be possible even in non-verbal children with severe ASD (Goh et al., 2013). To our knowledge, this is the only computer-assisted intervention which has been used so far in non-verbal children with ASD.

Indeed, to date, only few computer-based interventions are used in teaching literacy skills and academic skills in children with ASD, and their effects are limited and inconsistent (see Ramdoss et al., 2011; Knight et al., 2013 for reviews).

To address to these limitations and to provide the opportunity to minimally children with ASD to improve their communication and their global literacy skills, we developed a serious game, named SEMA-TIC, whose approach for the learning of reading is based on non-phonological processes. SEMA-TIC aims at teaching the pre-requisites for reading, i.e., to identify words as logographs and to learn basic syntax, without focusing on phonemic awareness.

While a video game can be defined as “a physical or mental contest, played according to specific rules, with the goal of amusing or rewarding the participants” (Zyda, 2005), a serious game can be seen as a video game in which the designers have concealed an educational or training purpose (Stokes, 2005).

It has been observed that children with ASD show great interest in technology and computers, and this interest could be used as a mean to improve their engagement and concentration in academic activities (Williams et al., 2002; Tuedor, 2006).

Based on this assumption, SEMA-TIC has been conceived to provide playful aspects while learning literacy skills. It has been designed based on the following principles: (1) Minimally verbal school-aged children with ASD present spared non-verbal cognitive abilities (Courchesne et al., 2015), on which SEMA-TIC relies to teach literacy skills. (2) Individuals with ASD are extreme “systemizers” and prefer organized environments based on logical rules (Baron-Cohen, 2006). Reading consists in applying a complex code, composed by many logical rules accompanied by exceptions. SEMA-TIC thus contains many words supporting the different logical exercises-games to address literacy skills. (3) Individuals with ASD are atypical learners (Qian and Lipkin, 2011), for whom the storage of specific examples is easy, but generalization to other contexts is difficult. For this reason, SEMA-TIC includes exercises employing specific examples, but also contributing to transfer the newly-learned skills to other domains. (4) Individuals with ASD can rapidly learn new things, provided that they are given opportunity to do that (Mottron et al., 2006). Furthermore, it has been suggested that extensive exposure to educational materials might be more effective for individuals with ASD than typical forms of teaching (Kourkoulou et al., 2012). In this line, SEMA-TIC contains more than 5,000 words to promote literacy basic knowledge. (5) Children with ASD with spoken language limitations require appropriate interventions based on a non-phonological approach. Previous studies of our own group have shown that serious games without verbal instructions, such as JeStiMulE, can be beneficial for individuals with high but also low functioning autism (Serret et al., 2014). (6) Finally, individuals with ASD prefer to use new technologies for educational learning (Ramdoss et al., 2012; Allen et al., 2015; Lorah et al., 2015). Therefore, the environment developed in SEMA-TIC has been designed in order to increase the attractiveness of the game and the motivation to learn.

The main aim of the present work was to develop a specific intervention that employed a serious game (SEMA-TIC) to teach literacy skills to French minimally verbal school-aged children with ASD. The main purpose of the study was to investigate the usability of SEMA-TIC, defined as the degree at which a product can be used by identified users, its efficiency and effectiveness in allowing the target users to reach specific goals, as well as users' satisfaction in a specific context (Nielsen, 1994; Serret et al., 2014). Assessments thus addressed three specific domains of SEMA-TIC in French minimally verbal school-aged children with ASD:

1. Adaptability: are these children able to use SEMA-TIC, and is it engaging? (indeed, motivation is a key element for children with ASD)
2. Efficiency: are they able to progress in the games in a given time?
3. Effectiveness: does the training allow these children to improve the targeted abilities? We hypothesized that after SEMA-TIC training, a performance improvement would be found in literacy skills acquisition in the training group (minimally verbal school-aged children with ASD which used SEMA-TIC) compared to non-training group (minimally verbal school-aged children with ASD which did not use SEMA-TIC) using experimental literacy skill tasks (alphabet knowledge, word reading, word non-word discrimination, sentence reading and word segmentation).

The second purpose of this study, in relation with the effectiveness of SEMA-TIC, was to compare the results of the two ASD groups on standardized and validated French reading tasks (Alouette reading test, Lefavrais, 1965 and ODEDYS, Jacquier-Roux et al., 2002) before and after SEMA-TIC training.

## Methods

### Materials

#### SEMA-TIC game description

**Figure 1:**
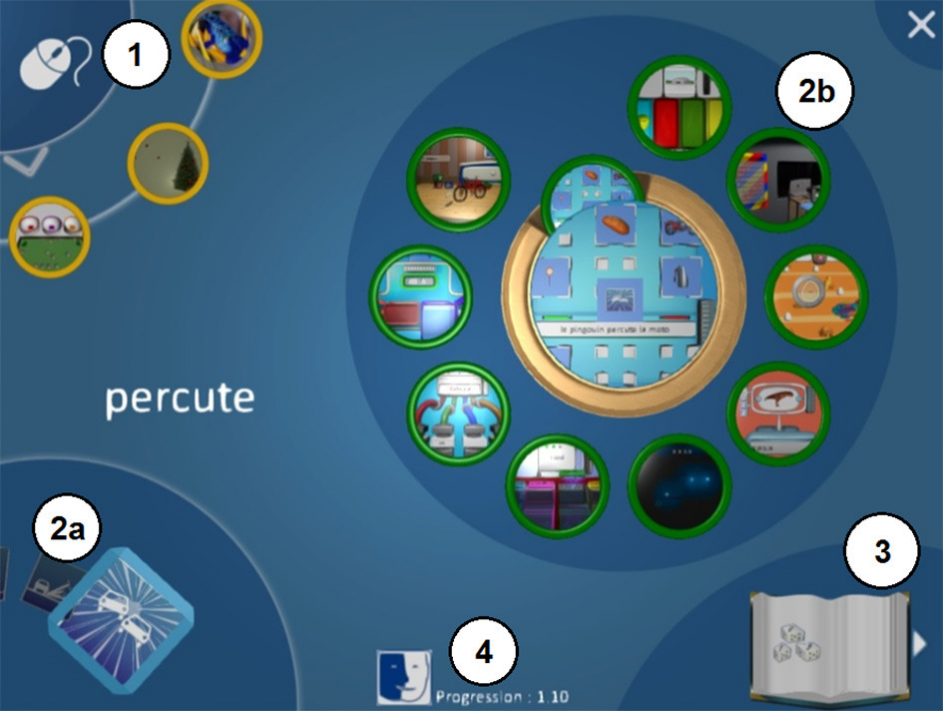
SEMA-TIC menu. (1) 3 mini-games for the player to learn how to use the mouse; (2a) selection of one of the 10 series (e.g., “hit”); (2b) selection of one of the 10 types of games; (3) dictionary of letters, words, and sentences; (4) monitoring for the caregiver.

SEMA-TIC includes (1) word-drawing associations, (2) sentence-3D animation associations (3) logical games with words and (4) logical games without verbal instructions (neither oral nor written). Its design allows children to discover the game using a trial and error strategy. SEMA-TIC was conceived as a tool to be used by a player accompanied by a trained caregiver and/or a family member. The caregiver and/or families can help the player either verbally, and/or through physical guidance, and/or through demonstration. SEMA-TIC includes a menu where players can discover SEMA-TIC's organization: 3 games designed to learn how to employ the mouse, 10 series of 10 games, a dictionary of letters, words and sentences and caregiver's monitoring (Figure 1). In order to verify the game reliability, during game's conception a user-test was conducted with reader children with ASD (*n* = 4) who used a prototype version of SEMA-TIC for 1 session; questionnaires were filled in by children's specialized educators, concerning the appropriateness of the game visual interface, the interest for the materials, the relevance of reinforcements, and the ease of use. Children and educators' feedbacks served as input to adapt game features during the game development process.

#### SEMA-TIC games

At the beginning of the scenario, 3 games allow the players to learn how to employ the mouse: (1) mouse drag, (2) point and click, and (3) point and drag-and-drop. After these 3 games, SEMA-TIC includes 10 series with 10 games each (for a total of 100 games) to teach literacy skills (Table 1). In all of these games, the player is asked to click on correct answers (words or images) or to drag and drop items to the correct locations (see Table 1 for a description of each game). Each game series employs a specific list of words, which is practiced in 10 different games.

**Table 1:** Description of the 10 games in SEMA-TIC, which are repeated in each series.

	**Games in each series**	**Learning**	**Literacy skills**	**Synthetic voice**	**Task**
1	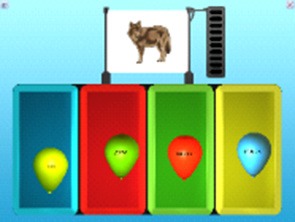	Word-drawing associations (9 nouns/1 verb)	Whole word recognition	Yes	Click on the balloon with the word corresponding to the displayed drawing
2	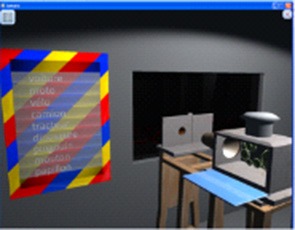	Word-multiple pictures associations (9 nouns/1 verb)	Whole word recognition (to generalize word recognition)	Yes	Pick a word from the list, and see different drawings corresponding to this word; then click on another picture displaying the word
3	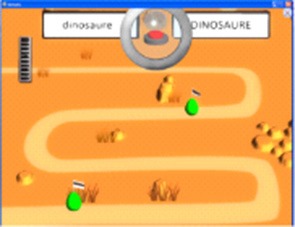	Upper-lower case associations of word (9 nouns/1 verb)	Alphabet knowledge	No	Among 10 items, click to form pairs of words displayed in upper and lower case
4	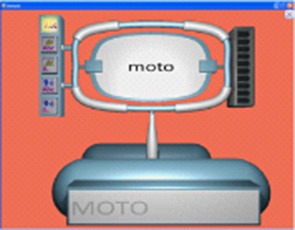	Writing words (9 nouns/1 verb)	Writing	Yes	Press keyboard keys to write the displayed words or images
5	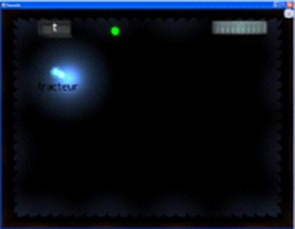	Searching words with similar first letter/visual syllable	Literacy convention	No	Click on the words beginning with the specified letter of syllable
6	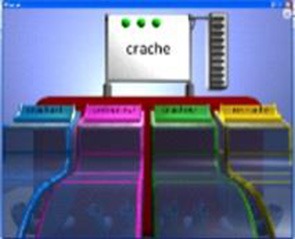	Searching words of the same family	Root words	No	Click on the words of the same family than the reference word
7	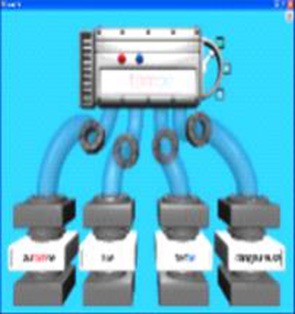	Create words with visual syllables included in other words	Word parts	No	Form the target word by clicking in the correct order on 4 words with colored syllables
8	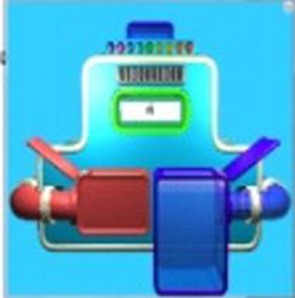	Word non-word discrimination	Letter co-occurrence inherent in French	No	Drag each item in the correct box, depending on if the item is a word or a non-word
9	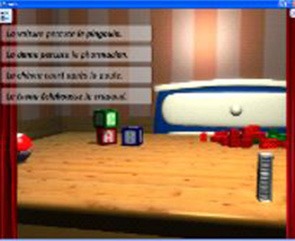	Reading a Sentence (9 nouns/1 verb)	Reading comprehension by visual action	Yes	Click to select the correct sentence, corresponding to the 3D visual animation (implying 2 words and 1 verb)
10	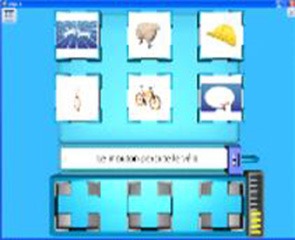	Sentences organization with subject-verb-direct object (9 nouns/1 verb)	Literacy convention	Yes	Drag and place the correct drawings in the correct order, to correspond to the displayed sentence

Stimulus presentation and game mechanics within a game are similar for all series. The difficulty of the games is progressively increased within a series, to promote learning. Multiple-choice questions (MCQ) are presented at the end of some games, to verify participants' acquisitions. Participants can progress to the next game after succeeding in 5 out of the 10 MCQ, and can progress to the next series after succeeding in all the 10 games of a series; the player is then proposed to train on another series, employing different words.

Reinforcements and feedback are provided during and after each game: A feedback is provided after each correct or incorrect response, so that the player can monitor his/her own performance, and visualize that thanks to a vertical colored gauge which fills up with correct responses, and gets emptier in case of incorrect responses. As a reinforcement, 3D video animations are displayed after a certain number of successful trials (the number of successful trials to obtain the reinforcement increases through the game progression, and after each game completion). These videos were created based on our clinical experience with children with ASD, and according to children's feedbacks during pre-tests. The videos consist in bright moving objects matching the interests of children with ASD, and allowing to maintain their attention (e.g., fireworks, bouncing balloon, etc.).

To summarize, these 10 series of 10 games propose a structured, progressive and adapted learning procedure based on nonverbal cognitive skills and without verbal instructions.

#### SEMA-TIC content

SEMA-TIC includes (1) 100 words (9 nouns or adjectives, and 1 verb in each of the 10 series), (2) 50 sentences employing these 100 words, and (3) more than 5,000 words used as distractors (in some of the games). All words were chosen from *Manulex*, a validated web-accessible database which provides grade-based frequency lists of the 1.9 million words found in French elementary school readings (Lété et al., 2004). The 100 words trained in the games were chosen according to a number of criteria: (1) word length between 2 and 10 letters, (2) words composed of 1–4 syllables, (3) words graphically displayable in pictures (for isolated words) and 3D animations (for sentences). The 100 words were allocated to each series to obtain series of balanced mean word lengths, number of syllables, word frequency in the French language, and by topic (in order to provide coherent sentences using the words in each series). Furthermore, the words were chosen according to the interests of children with ASD, and to their usefulness in daily life. Finally, the choice of the words and sentences was validated based on an inter-professional agreement, based on focus groups including speech therapists, psychologists, teachers and parents of children with ASD. The properties of the 100 selected words trained in the series are provided in Table 2.

**Table 2:** Properties of words used in each series.

**Series**	**Number of letters**	**Number of syllables**	**Frequency per million**
1	6.7 (1.7)	2.5 (0.7)	216.4 (258.3)
2	6.2 (1.6)	2.2 (0.9)	155.7 (199.6)
3	6.2 (1.7)	2.2 (0.9)	166.8 (154.8)
4	7.4 (2)	2.3 (1.1)	190.8 (451.6)
5	8.3 (1.9)	2.8 (0.8)	89.3 (98.5)
6	6.2 (2)	1.9 (0.7)	236.1 (194.1)
7	5.1 (1.8)	1.8 (1)	1,252.2 (3,079.1)
8	4.7 (1.2)	1.8 (0.6)	280.4 (351.9)
9	5.5 (1.3)	1.7 (0.7)	455.2 (510.4)
10	6.5 (1.4)	2.4 (0.5)	148.5 (114)
Total	6.2 (1.9)	2.1 (0.8)	318.8 (1,014.3)

*Mean and standard deviation for number of letters, number of syllables, and frequency per million in first-grade class books. It has to be noted that the high mean frequency is caused by the presence of the verb “is” in this series, which is of course very frequent*.

#### Trained literacy skills in SEMA-TIC

SEMA-TIC targets the training of literacy skills through 3 categories of games.

**Figure 2:**
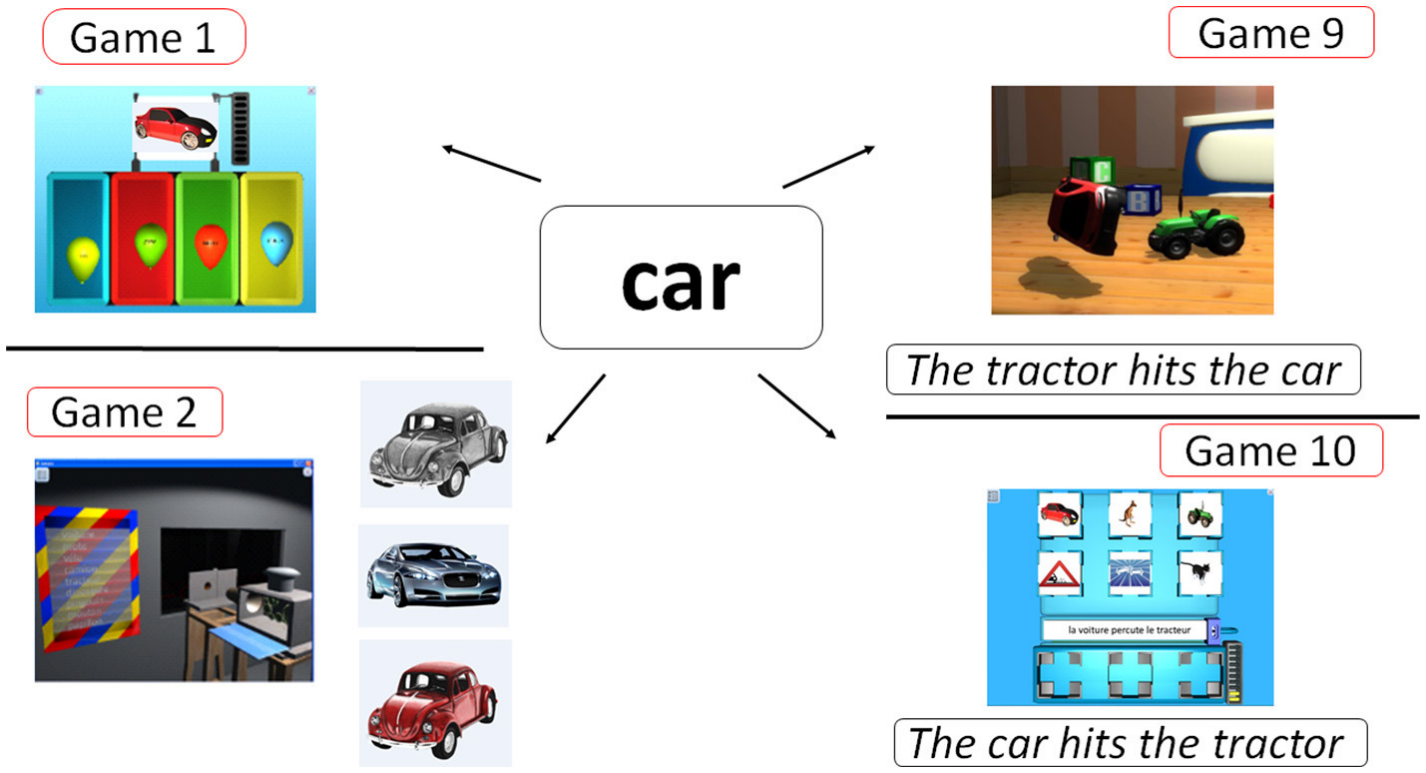
SEMA-TIC games implying whole word recognition and learning association. In Game 1, the player has to choose the correct word, based on the its verbalization and image. In Game 2, the player is shown several copies of an object to generalize his vocabulary. In Game 9, the player has to choose the correct sentence, based on a 3D animation implying 2 objects and 1 verb. In Game 10, the player has to choose correctly and put in order 3 pictures, based on a written sentence.

The first category of games concerns whole word recognition and word learning association (Figure 2), and includes vocabulary learning by word-drawing associations employing a synthetic voice (Game 1), vocabulary generalization by word-sketches-pictures associations employing a synthetic voice (Game 2), sentence understanding promoted by a visual action with 3D animation employing synthetic voice (Game 9) and making simple sentences with subject-verb-direct object (Game 10).

**Figure 3:**
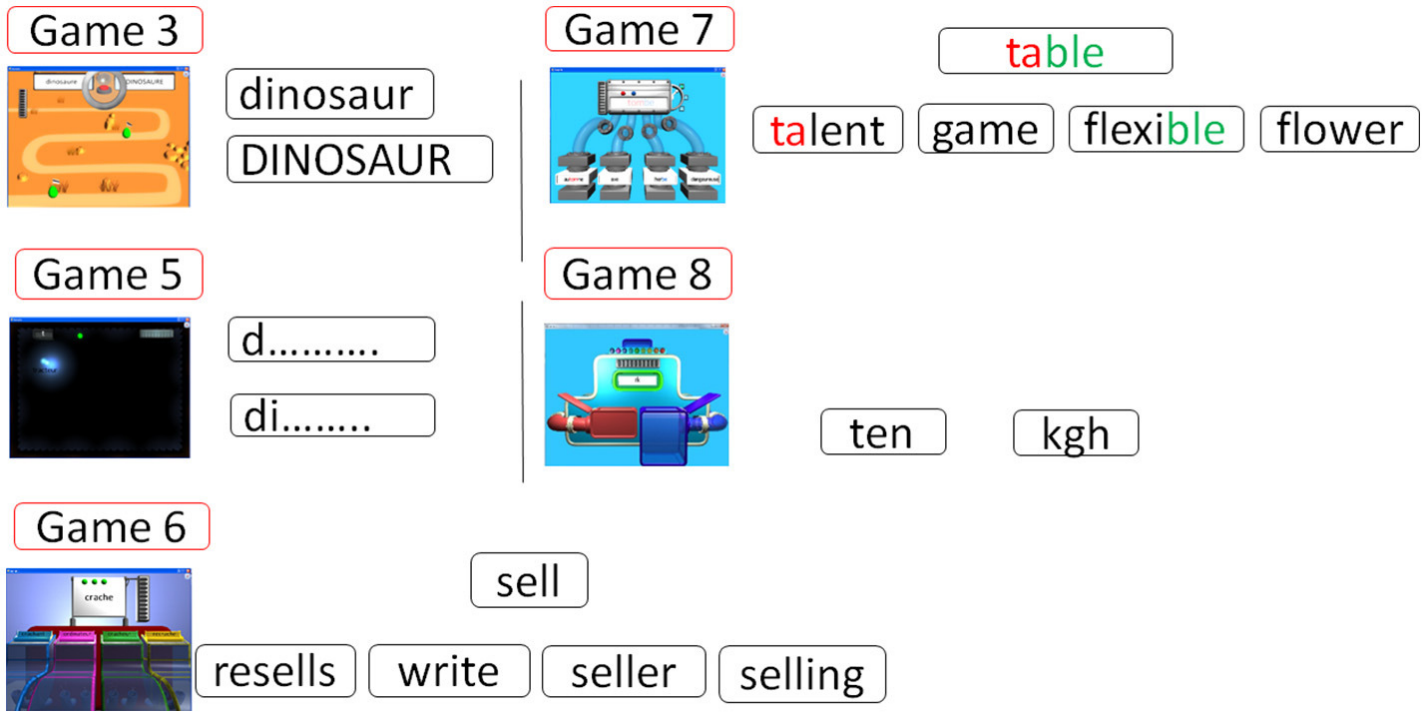
SEMA-TIC games implying alphabet knowledge and decoding. In Game 3, the player has to pair words written in uppercase and in lowercase. In Game 5, the player has to identify words correctly according to their first letter or syllable. In Game 6, the player has to choose correct words based on families. In Game 7, the player has to select words to form a new word from some visual syllables. In Game 8, the player has to decide if a series of letters could exist or not in the French language.

The second game category targets alphabet knowledge and decoding (Figure 3), and includes lower-upper case associations (Game 3), selecting the correct words on the screen according to their first letter and their first visual syllable (Game 5), identifying word families (Game 6), making words from visual syllables (Game 7) and words/non-words discrimination (Game 8).

**Figure 4:**
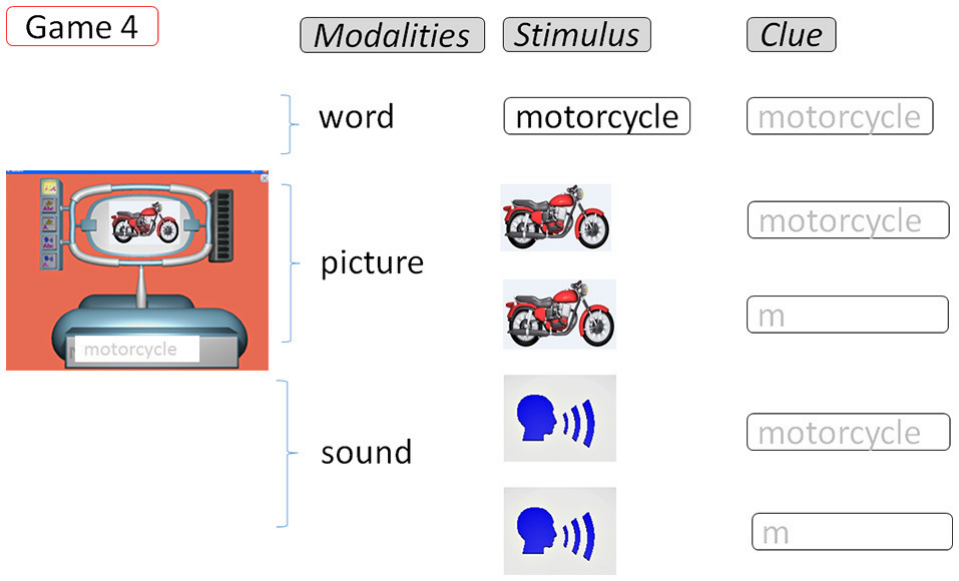
SEMA-TIC game implying writing. In Game 4, the player has to type on the keyboard a given word after the presentation of this word, a picture, or an oral verbalization. All letters to be typed can remain displayed, or only the first one.

The last game category concerns writing (Figure 4), with 5 different tasks consisting in typing a given word after the presentation of this word, a picture representing this word, or an oral verbalization of this word, combining with 2 possible levels of clueing (Game 4).

### Participants

Thirty ASD children with verbal limitations were initially recruited in local day-care units by the Autism Resources Center of Nice (University Child and Adolescent Psychiatry Department). Inclusion criteria were (1) a diagnosis of ASD based on DSM 5 (American Psychiatric Association, 2013) criteria, as well as on the Autism Diagnostic Interview-Revised (ADI-R) (Rutter et al., 2003) and/or the Autism Diagnostic Observation Schedule (ADOS) (Lord et al., 2000); (2) age from 6 to 11 years; (3) a level of verbal communication based on item 11 of Childhood Autism Rating Scale-Translated (CARS-T) ≥ 3 (Schopler et al., 1988); (4) analogical reasoning ≥ 15 (raw score) using Raven's Colored Progressive Matrices board form (RCPM) (Raven et al., 1998), corresponding to the average score of 5 years old children, to ensure that participants had sufficient reasoning skills to allow learning in SEMA-TIC; (5) absence of formal reading instruction experience; (6) no reading skills assessed on validated reading tests (Alouette reading test, Lefavrais, 1965 and ODEDYS, Jacquier-Roux et al., 2002); (7) minimally verbal according to the definition of Kasari et al. (2013) and using two items of the Evaluation Language Oral test (ELO) (Khomsi, 2001), with repetition and production of utterance ≤3 years.

Two ASD groups were constituted. Fifteen children were included in the training-group and employed SEMA-TIC. Due to logistic constraints, the control group was constituted after the beginning of the intervention, and included 15 children who did not use SEMA-TIC. Each child in the non-training group was matched by age to the training-group participants. No intervention was provided to the children in the non-training group, as the purpose of this preliminary study was to investigate the contribution of the SEMA-TIC intervention compared to usual functioning.

Of the 30 participants initially recruited, five did not complete the study protocol for different reasons: two children in the non-training group did not come for the final evaluation; parents of two children in the training group did not accept to use SEMA-TIC at home after the beginning of the study; one child in the training group and his family moved out (see Supplemental File 5 for dropped-out participants characteristics). These five children were excluded from data analyses. In total, 25 children with ASD completed the study, with 12 participants in the training-group and 13 participants in the non-training group. The participants' characteristics are presented in Table 3 (see also Supplemental File 2). There was no significant difference between the two groups on any clinical feature.

**Table 3:** Training and non-training groups' clinical characteristics at inclusion (ASD, Autism Spectrum Disorder; M, Mean; SD, Standard Deviation).

**Groups**	**Training group**	**Non-training group**	***p*-value**
Number of participants	12	13	
ASD (DSM-5)	12	13	
Gender (M, male; F, female)	11 M, 1 F	10 M, 3 F	0.31
	***M*** **(*****SD*****)**	***M*** **(*****SD*****)**	
Age (years, months)	8.7 (1.8)	8.5 (1.8)	0.78
Intensity of autism (CARS-T)	39 (4.1)	37.8 (2.9)	0.4
RCPM (raw score)	20.1 (4.3)	21.2 (3.9)	0.51
Production of utterance (ELO) Mean (SD) for age 3	−1.1 (0.8)	−1.3 (0.7)	0.51
Repetition of utterance (ELO) Mean (SD) for age 3	−1.2 (0.6)	−1.4 (0.5)	0.31
Reader's abilities (ALOUETTE/ODEDYS)	0%	0%	1

### Setting

After inclusion, a meeting was organized with an experimenter, the caregiver, parents and child to provide an overview of SEMA-TIC game and study's procedures. Each child in the training-group was supervised by a trained caregiver, whose role was to guide the child in his progression in the games and in the series, and to validate the progression from a game to another. Parents' role was to practice the games already done with the caregiver, in order to consolidate learning. Caregivers and parents thus received a specific training about the games mechanics and procedures. SEMA-TIC was installed on a computer in the educational structures of the children (medical education institutes, or specialized home education and healthcare services) and at home with their parents. All training sessions in the educational structure were performed with a caregiver in a quiet room. Training sessions at home were performed with a trained parent. Compliance at home was verified by using home liaison diaries.

### Assessments

#### Adaptability assessment

Adaptability assessment included (1) the ability to use mouse (2) the ability to use the visual menu, (3) motivation to play. After the SEMA-TIC training was completed, each caregiver completed a brief questionnaire to collect information on the perceived acceptability of the intervention by the children. The questionnaire included a 5-point Likert-type scale for 3 questions. The questionnaire asked the caregiver to indicate their level of satisfaction (0 = *not at all* to 4 = *very satisfying*) concerning their perception about the ability of the child to use a mouse, the ability to use the visual menu of SEMA-TIC, and the motivation of the child to play SEMA-TIC.

#### Efficiency assessment

Efficiency assessment was defined as the time required by participants to complete each series once they had learned the game design.

#### Effectiveness assessment

Finally, the evaluation of effectiveness included the performance assessment on experimental tasks and standardized reading tests in both groups, before and after SEMA-TIC training. Participants were tested individually by an independent psychologist in a quiet room with pauses, if necessary.

##### Experimental literacy skill tasks

The experimental tasks were composed of words seen during the SEMA-TIC training as well as new words, never seen in SEMA-TIC. Four tasks were similar to tasks used in the training: (1) alphabet knowledge (upper-lower case associations), (2) word reading (word-drawing associations), (3) word-nonword discrimination (words with 2–10 letters), (4) sentence reading (short sentences with subject-verb-direct object). One additional task was created in order to evaluate the transfer of literacy skills to new tasks, and consisted in word segmentation (making separations in a group of letters to form 3 distinct words), thus implying alphabet knowledge and decoding. Two blocks of experimental tasks were scheduled at baseline (pre-test/T0) and after SEMA-TIC training (post-test/T1). To avoid children's frustration, evaluation tasks at baseline only used material of the first series of SEMA-TIC. Evaluation tasks conducted after SEMA-TIC training used material from all 10 series of SEMA-TIC, thus with 10 times more items. All five experimental literacy skills tasks are described in Table 4 (see also Supplemental File 1).

**Table 4:** Description and contents of the 5 experimental tasks used for the assessment of literacy skills.

**Experimental tasks**	**All tasks are presented on pictures handle format**	**Materials seen on SEMA-TIC**	**Materials not seen on SEMA-TIC**
Alphabet knowledge	1 uppercase word is presentedChoose 1 among 4 lowercase words	/5 items (upper-lower cases associations with SEMA-TIC words)	/5 items (upper-lower cases associations with new words)
Word reading	1 drawing is presentedChoose 1 among 4 written words1 written word is presentedChoose 1 among 4 drawings	/20 items (words-drawing SEMA-TIC associations)	/10 items (SEMA-TIC's words-pictures associations)
Word nonword discrimination	1 word or 1 non-word is presentedParticipants have to put the word in a blue box and the non-word in a red box	/10 items (SEMA-TIC's words)	/30 items (words not seen in SEMA-TIC)
Sentence reading	1 written sentence is presentedChoose 1 among 4 pictures1 picture is presentedChoose 1 among 4 writing sentences	/10 items Sentences learned in SEMA-TIC	/10 items Sentences not learned in SEMA-TIC/5 New sentences (one out of three sentence's words were unknown)
Word segmentation(New task unseen on SEMA-TIC)	3 words to be segmented with a pen	/10 items	/5 items

In order to verify the task's reliability, a preliminary data collection was conducted in a group of typical developing children (*n* = 16) at the end of first grade of primary regular French school. Results are presented in Supplemental File 2.

##### Validated reading tests

Standardized reading tasks included two French classical validated tests: (1) the Alouette Reading Test, in which children have to read 5 letters, 10 words, and a text of 265 words which they have to read in 3 min and which includes familiar and unfamiliar words; the test structure was built to avoid lexical knowledge-based guessing (Lefavrais, 1965); (2) the ODEDYS, in which participants have to read 60 isolated items, consisting of 20 regular words, 20 irregular words and 20 pseudo-words (Jacquier-Roux et al., 2002). The Alouette Reading Test has been shown to have a satisfying discriminatory power in distinguishing dyslexic readers from non-dyslexic readers in young adults, with 83.1% sensitivity and 100% specificity (Cavalli et al., 2017). Unfortunately, to our knowledge no psychometric data exists concerning the ODEDYS.

### Study procedure

After inclusion, all participants were tested on standardized and experimental tasks at baseline, 2 weeks before training. All training sessions of the training-group were conducted with a trained caregiver in the educational structure, and with parents at home. Each week had to be composed of 4 h of training (approximately 2.5 h in the educational structure and 1.5 h at home), although the number and duration of the sessions within some specific weeks had to be adapted for each child (because of life events such as diseases, holidays, field trips). Participants played until they completed SEMA-TIC. Finally, all participants were tested on experimental and standardized tasks 2 weeks after SEMA-TIC training. Game data were collected by the caregiver of each participant. All children continued to follow their individual and institutional interventions (such as speech therapy and behavioral and developmental interventions) as well as special educational pre-school learning during the overall study.

All procedures were approved by the Local Ethical Committee (Comité de Protection des Personnes Sud Méditerranée V: reference number 13.046, accepted 8th October 2013) and by the French National Security Agency for Medicines and Health Products Safety (ANSM), (trial registration number: 2013-A01024-41 accepted July, 24th 2013). Written informed consent was obtained from all participants and their parents prior to inclusion in the study, in accordance with the Declaration of Helsinki.

### Statistical analyses

Descriptive statistics (mean and standard deviation) were calculated for participant's clinical characteristics. Student's *t*-test was performed in order to test for statistical differences between groups for these variables at baseline and after the end of the intervention, at the two-sided significance level of *p* < 0.05. Because baseline and post-test evaluations had different number of items, we did not analyze statistically the difference between pre- and post-test performance. We hypothesized that while the performance of the training and the non-training group would be similar at baseline, the training group should significantly improve in the experimental literacy skills tasks after the intervention, with no improvement in the non-training group. Standardized tasks evaluations were described with raw scores and standard deviations according to normative data for second grade of regular school on typical readers.

## Results

### Results on adaptability

Results of the questionnaire suggested that the caregivers judged that 100% of the participants of the training-group were able to use the mouse to play SEMA-TIC. They were also 100% capable of making a correct choice in the visual menu. Strong motivation to play was found in 75% of the participants (see Table 5).

**Table 5:** Results of SEMA-TIC adaptability.

**Users abilities**	**% of participants (*n*)**
Adequate use of the mouse	100% (*n* = 12)
Adequate use of the visual menu	100% (*n* = 12)
Strong motivation to play	75% (*n* = 9)

### Results on efficiency

Results showed that ASD training group completed SEMA-TIC after 23.6 weeks (*SD* = 7.2; range: 15–36 weeks). The completion of first series was achieved after a mean of 4.3 weeks of practice. The series 2, 3, and 4 were completed on average after 6.3 additional weeks; the series 5, 6, and 7 after 6.6 weeks, and the series 8, 9, and 10 after 6.8 weeks.

See Table 6 for details on the individual progression in the game series.

**Table 6:** Individual performance scores in the experimental tasks for the both groups in pre- and post-tests, and time progression in SEMA-TIC series, expressed in the number of weeks needed for each participant since the beginning of the intervention to reach and achieve the indicated series.

	**Pre-test (% performance)**					**Series (weeks)**				**Post-test (% performance)**				
**Training group**	**AK**	**WR**	**WNWD**	**SR**	**WS**	**S1**	**S4**	**S7**	**S10**	**AK**	**WR**	**WNWD**	**SR**	**WS**
1	0	0	15	0	0	4	6	11	18	100	70	95	60	60
2	0	10	10	0	0	4	15	22	31	70	80	90	32	38
3	80	70	25	6.7	2	6	8	11	15	100	100	97.5	100	75.3
4	0	3.3	15	0	0	4	17	26	36	100	53.3	52.5	52	20
5	60	35	0	13.3	0	3	9	12	16	100	100	90	100	95.3
6	0	0	30	20	8.7	4	9	15	20	100	100	100	100	93.3
7	0	0	20	13.3	0	5	13	19	27	100	96.7	95	88	0
8	60	3.3	0	0	0	5	8	22	32	100	70	62.5	60	4
9	60	25	15	13.3	4	4	13	16	20	100	100	85	100	46.7
10	0	0	17.5	0	0	4	9	14	15	100	50	67.5	40	0
11	0	0	10	0	0	4	11	16	27	100	96.7	90	92	46.7
12	0	3.3	0	13.3	0	4	9	22	27	100	83.3	87.5	76	28.7

### Results on effectiveness

#### Experimental literacy skill tasks

**Figure 5:**
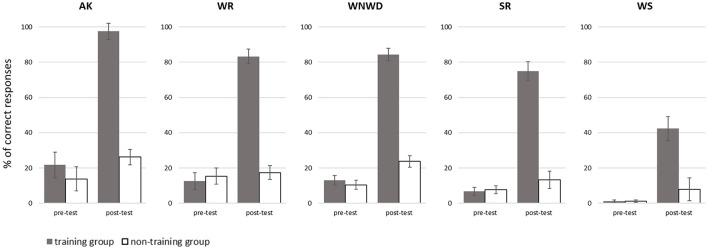
Evolution of performances in the experimental tasks. Results of training group (gray bars) compared to non-training group (white bars). Mean percentage of correct responses before (pre-test) and after (post-test) intervention, with standard deviations, for each experimental task (Alphabet Knowledge AK, Word Reading WR, Word Non-Word Discrimination WNWD, Sentence Reading SR, and Word Segmentation WS).

There was no significant difference between the two groups on experimental tasks at baseline (all *p* > 0.05, see Table 7), indicating similar literacy skills in both groups of children with ASD involved in this study. However, in post-test, performance scores significantly differed between the 2 groups for each experimental task (all *p* ≤ 0.001, see Table 7), indicating that children with ASD in the training-group had significantly better performance in literacy skills in these experimental tasks compared to children in the non-training group (Figure 5). Interestingly, the significantly higher performance in the training-group was also observed in the segmentation task, even though this task was not trained in SEMA-TIC (see Table 6 for the individual performance progression in the training group).

**Table 7:** Results of SEMA-TIC effectiveness.

	**Before SEMA-TIC training (pre-test/T0)**				**After SEMA-TIC training (post-test/T1)**			
**Participants**	**Training group**	**Non-training group**	***t*_(23)_ value**	***p*-value**	**Training group**	**Non-training group**	***t*_(23)_ value**	***p*-value**
Alphabet knowledge	21.7 (7.2)	13.8 (6.9)	0.78	0.44	97.5 (4.6)	26.2 (4.4)	11.29	<0.001
Word reading	12.5 (4.9)	15.4 (4.7)	−0.43	0.67	83.3 (4.2)	17.4 (4)	11.29	<0.001
Word non-word discrimination	13.1 (2.7)	10.4 (2.6)	0.72	0.48	84.4 (3.5)	23.7 (3.3)	12.65	<0.001
Sentence reading	6.7 (2.3)	7.7 (2.2)	−0.33	0.75	75 (5.2)	13.2 (4.9)	8.62	<0.001
Word segmentation	1.2 (0.6)	1.2 (0.6)	−0.01	0.99	42.3 (6.9)	7.9 (6.6)	3.6	<0.001

*Performance scores (mean and standard deviation) in the experimental tasks for the training and non-training groups, in pre- and post-tests, with T-test and probability values*.

#### Validated reading tests

Results showed that before the intervention, none of the children with ASD could decode efficiently regular words, irregular words or pseudo-words in the ODEDYS and the Alouette reading tests. Indeed, all participants scored 0 in all items of the validated reading tests at baseline. After SEMA-TIC training, 3 out of the 12 children with ASD in the training-group participants developed efficient decoding skills as measured by these reading tests. Indeed, these 3 children had the maximum score in reading isolated letters (5/5) and isolated words (10/10) in the Alouette reading test, and were able to read correctly more than 30 words in a text composed of 265 words (see Table 8 and Supplemental File 4 for individual results). They were also able read correctly a certain number of irregular words and pseudo-words, thus showing a clear improvement in their decoding skills, although some of these individual scores were below scores from typical readers in second grade of regular school, as indicated by normative data (see Table 8). Other children in the training-group only showed slight improvements in reading isolated letters (mean score: 3.3 words; *SD* = 2.5). More importantly, the significant improvements were specific to the training-group, as only very slight improvements were observed in this group in reading isolated letters (mean score: 2.5 words; *SD* = 2.4) and in reading isolated words (mean score: 0.4 words, *SD* = 0.87).

**Table 8:** Results on standardized reading tests for participants 3, 6, and 9.

**a-Alouette reading test**	**Number of letters read (/5)**	**Number of isolated words read (/10)**	**Number of words read in text (/265)**	**Number of errors**	**Number of words read correctly**	**Readability index**	**Reading speed index**
Participant 3	5	10	36	1	35	97.22 (+1 *SD*)	35 (−2 *SD*)
Participant 6	5	10	83	4	79	95.18 (0 *SD*)	79 (0 *SD*)
Participant 9	5	10	52	14	38	73.07 (−2 *SD*)	38 (−1 *SD*)
**b-ODEDYS**		**Regular words (/20)**		**Irregular words (/20)**		**Pseudo-words (/20)**	
Participant 3		15 (−1 *SD*)		13 (−1 *SD*)		15 (0 *SD*)	
Participant 6		19 (0 *SD*)		10 (−2 *SD*)		18 (+2 *SD*)	
Participant 9		8 (−2 *SD*)		2 (−2 *SD*)		11 (−2 *SD*)	

*Results on Alouette Reading Test (a) and ODEDYS (b) on the 3 readers of the training group after SEMA-TIC training. Results provides raw scores and standard deviations according to normative data for second grade of regular school*.

## Discussion

### Summary

The present exploratory study aimed at investigating SEMA-TIC usability on literacy skill acquisition in French minimally verbal school-aged children with ASD, in terms of adaptability, efficiency and effectiveness.

Adaptability assessment aimed at investigating if users were able to use SEMA-TIC, and if they found the games engaging. Results provided evidence that children with ASD were able to handle correctly the computer mouse and to navigate through game menus, and thus to interact appropriately with games mechanics, highlighting their ability to learn new competence in an appropriate learning context. Moreover, one important result concerned children's motivation to play SEMA-TIC. Indeed, according to caregivers, most of the children in the intervention group expressed interest and investment in SEMA-TIC games, expressed satisfaction about items and gameplays of the different games, and showed great interest in the reinforcement videos. Children were reported to produce less stereotyped behaviors, to be less agitated, and to stay longer in front of the computer screen, when playing SEMA-TIC. In line with the principles of a serious game, the strong interest in playing SEMA-TIC observed in our participants confirmed the possibility for children with ASD to access academic skills learning procedures through a fun and computerized system. The children were thus involved in playing the games while promoting the acquisition of knowledge (Stokes, 2005).

SEMA-TIC was conceived for children with ASD with verbal limitations, and thus does not include verbal instructions, so that users with ASD have to deduce games rules by trial-and-error strategies as well as by the repetition of the same games (e.g., similar games throughout the 10 series). MCQ used to validate acquisition during games indirectly allowed to ensure the understanding of games instructions during the progress in SEMA-TIC, indicating that minimally verbal children with ASD played efficiently.

Concerning the efficiency of SEMA-TIC, the number of training sessions varied from one participant to another, according to individual learning rates. However, all participants achieved the first series after an average of 4 weeks, while they achieved each following series after about 2 weeks, suggesting a better understanding of the games rules and mechanics after an initial discovery phase of SEMA-TIC. These results thus indicate that children in the intervention group were actually able to progress in the games series.

Remarkably, results concerning effectiveness showed that minimally verbal school-aged children with ASD in the intervention group were able to acquire efficient literacy skills and, most importantly, to transfer their learning to novel materials, as observed in the results on the experimental tasks. They were not only able to memorize SEMA-TIC materials (word and sentence reading tasks), but also to apply simple rules (alphabet knowledge task) as well as complex rules (word non-word discrimination task) taught in SEMA-TIC to new material. In addition, most of the children were able to transfer the knowledge learnt during the training to a novel task, which was not trained (the word segmentation task) and involved alphabet knowledge, literacy conventions, and decoding skills. Moreover, 3 out of the 12 trained participants achieved clinically significant improvements in validated reading tests after SEMA-TIC training, and could thus be considered as efficient word decoders following the study period according to these validated tests (although their performance remained below scores from age-related typical reader children). This result could correspond to an efficient transfer of learning in some of the SEMA-TIC users. It should be noted that two of these children had better spoken language performance than the other children of the training group at inclusion (participants 6 and 9, see Supplemental File 3). However, their spoken language level was actually low, as it corresponded to the production of utterance of 3 years old typically developing children, whereas these two children were, respectively, 6.2 and 11.4 years old. Moreover, participant 3 (7 years old) significantly improved his literacy skills after the training and could became considered as efficient word decoder, despite his spoken language level was significantly below the production of utterance of 3 years old typically developing children at inclusion. Thus, improvements in literacy skills could not only be attributed to the initial level of language.

In addition, none of the children who did not train on SEMA-TIC improved in the validated reading tests and could be considered as an efficient decoder after the study period.

Taken together, we thus consider these observations as clinically significant evidence for the effectiveness of SEMA-TIC.

SEMA-TIC aims at promoting basic literacy knowledge of the reading code, and could therefore represent an alternative to the traditional phonics approach. In consequence, it might pave the way for a literacy skills instruction program for minimally verbal French children with ASD, who are often excluded of formal reading instruction at regular school. The 10 SEMA-TIC games were designed based on children with ASD's non-verbal cognitive abilities, including (1) visual memorization, to memorize whole words and sentences, (2) focused attention, for being attentive on details while writing and recognizing words with similar first letter/visual syllable, in order to discover literacy conventions, (3) analogical reasoning, to learn alphabet, root words and the sequence of visual syllables composing words, in order to discover the logical rules of visual reading code, and finally (4) implicit learning, to discover letter co-occurrence inherent in the French language. All these non-verbal cognitive skills, frequently underestimated in minimally verbal children with ASD, have been used to develop specific learning strategies, which appeared clinically efficient in the children involved in this study.

In contrast to most of the studies conducted in this field, which focused on the training on sight word recognition in children with ASD with significant cognitive disabilities and verbal limitations (Chiang and Lin, 2007; Spector, 2011; Ramdoss et al., 2012), SEMA-TIC offers more broadly and diversified learning techniques, including visual and logical approaches with letters, visual syllables, words and sentences, in order to promote the learning to read as a visual code solving. To our knowledge, only very few studies addressed literacy skills in minimally verbal children with ASD or the improvements in reading through a nonverbal intervention, while no study so far addressed these two thematic together. Our results are in line with the study of Goh et al. (2013), which also focused on the training of literacy skills in children with ASD with verbal limitations. Their study included a computerized intervention and similarities in the training, implying nonverbal abilities (i.e., visual sequencing of letters in words, of words in sentences, and noun-verb pairing). Despite important drop-out rates, their results showed significant improvements in experimental tasks of literacy skills. However, to our knowledge, our study is the first to show improvements in validated reading tests in addition to experimental tasks. In line with nonverbal interventions targeting reading skills, a recent study showed that playing action video games (not linked to language or reading) could improve reading skills in dyslexic children, thanks to spatial and temporal attention improvements during the training (Franceschini et al., 2013). Taken together, these results represent growing evidence for the relevance of targeting nonverbal skills through computerized interventions to improve literacy and reading skills in developmental disorders.

In summary, our study indicates that SEMA-TIC presents a suitable usability, as all participants were able to play appropriately and to engage in the training (adaptability), to progress in the series and to complete the training (efficiency), and to acquire and improve some literacy skills (effectiveness). The design of SEMA-TIC, which relies on non-verbal cognitive skills to teach verbal skills and implies atypical learning strategies, seems thus to be adapted to minimally verbal school-aged children with ASD for the training of literacy skills. Overall, the results of our exploratory study suggest that French minimally verbal school-aged children with ASD are able to understand, to learn and to be evaluated by means of experimental and standardized tasks upon a specific intervention adapted to this population.

SEMA-TIC appears to provide a great opportunity for children with TSA to access to the learning of literacy skills, and could be used as an alternative to traditional phonics methods. Indeed, several minimally verbal children with TSA became effective decoders through their use of SEMA-TIC, which allowed their caregivers to propose more diversified procedures for learning to read and to address reading comprehension skills. The use of SEMA-TIC also encouraged the teachers to use this serious game with children who were not included in the study.

Finally, SEMA-TIC has shown evidence to the caregivers that learning through new technologies allow faster, greater and more motivating learning.

## Limitations

Although very encouraging, our results should be considered with great caution, as the current study was exploratory.

A first important limitation is the absence of randomization between the two groups of children with ASD, as the non-training group could only be constituted after the beginning of the study. Although the initial purpose of our study was to evaluate the usability of SEMA-TIC with minimally verbal school-aged children with autism, a randomized controlled study with homogeneous groups matched on cognitive abilities would provide stronger evidence concerning the efficiency of the training. Moreover, in the present study, the control group did not receive any intervention. Further studies will require addressing this limitation, and proposing alternative interventions to the participants in the control group.

Another limitation of this study is the definition of the population of minimally verbal school-aged children with ASD, as this definition does not take into account other cognitive abilities (i.e., non-verbal skills). Indeed, our population was recruited based on limited verbal skills but also based on higher non-verbal cognitive skills. Detailed characterization of the children's non-verbal skills would allow a better understanding of the scope and of the efficiency of each game, considering that each game relies on a different combination of non-verbal skills.

Furthermore, this study concerned the learning of literacy skills in the French language, thus requiring further replications in other languages before generalizing conclusions.

### Perspectives

Future researches will need to further explore the potential of SEMA-TIC and should focus on (1) investigating precisely the number of sessions needed for efficient learning in each exercice; (2) identifying the cognitive skills in children with ASD which are the most important to efficiently learn literacy skills and to transfer learnings; (3) evaluating the usability of SEMA-TIC in regular classrooms and institutions; (4) assessing the efficacy of SEMA-TIC compared to formal reading instruction programs based on phonological approaches, as well as its association with other supports of reading skills learning (i.e., pictures, books, computer-based applications). SEMA-TIC exercices and mechanics would significantly benefit from these researches, and would thus enhance their learning potential.

Language processing difficulties limit the psychosocial opportunities of intellectually able autistic people (Howlin et al., 2004). Previous research suggests the existence of links between reading components teaching and language development such as oral vocabulary acquisition (Cain et al., 2004; Ricketts et al., 2015) and between the cognitive process of writing and augmentative communication (Koppenhaver and Williams, 2010). SEMA-TIC helped children with ASD in learning and memorizing word-picture associations (game 1 and 2) and in writing words (game 4), but vocabulary and communication were not assessed in our study. It would be interesting for future research to investigate if playing SEMA-TIC contributes to improve vocabulary and communication in minimally verbal school-aged children with ASD.

SEMA-TIC enabled the access to academic knowledge such as literacy skills, using specific learning strategies based on non-verbal cognitive skills. We suggest that other academic domains, such as mathematics, could be teached in the same way. Indeed, a recent study showed that the strongest predictor of math problem solving was perceptual reasoning, which is a non-verbal cognitive skill (Oswald et al., 2016). Interestingly, the ability to solve applied mathematical problems is associated with everyday problem-solving abilities and vocational outcomes. Then, it could be interesting to investigate the improvements in problem solving and in the understanding of daily living situations after literacy and mathematical skills training through non-verbal methods.

## Conclusions

Reading instruction is as an important academic skill, typically learned at school. It promotes access to knowledge for the developing child, and promotes his inclusion in the society. Each child deserves the best instruction possible to develop his ability to read at the highest possible level. Spoken language limitations and communication impairments in minimally verbal school-aged children with ASD significantly reduce their academic learning exposure at school. SEMA-TIC proposes to these children a specific and adapted basic literacy skills exposure. Indeed, this study provided further evidence that teaching literacy skills to children with ASD is not ineluctably linked to spoken language.

The findings of our exploratory study suggest that minimally verbal school-aged children with ASD are able to learn literacy skills through a different way compared to typically developing children. Therefore, our results pave the way for novel educational approaches based on theoretical support which has been mostly unexplored in the past: an academic instruction based on visual and logical approaches.

## Author contributions

SS, SH, ST, PP, AS, and FA contributed to the conception and design of the study. SH, PP, and AS contributed to data acquisition. SS and JB contributed to data analysis and interpretation. All authors contributed to the draft and the revision of the present paper, approved the version to be published, and agreed to be accountable for all aspects of the work in ensuring that questions related to the accuracy or integrity of any part of the work are appropriately investigated and resolved.

### Conflict of interest statement

The authors declare that the research was conducted in the absence of any commercial or financial relationships that could be construed as a potential conflict of interest.

## Supplementary material

Supplemental File 1 Example of the items used in the experimental tasks. All materials have been translated to English for this paper. **(A)** Alphabet knowledge, **(B)** Word reading, **(C)** Word/non-word discrimination, **(D)** Sentence reading, **(E)** Word segmentation.

Supplemental File 2 Results of SEMA-TIC experimental tasks for typical developmental children (*n* = 16).

Supplemental File 3 Training and non-training groups' clinical and cognitive characteristics detailed at inclusion.

Supplemental File 4 Individual performances in validated reading tests.

Supplemental File 5 Drop-outs' clinical and cognitive characteristics at inclusion, and performances at standardized and experimental tasks in pre-test.

## Abbreviations

ADI: Autism Diagnostic Interview-Revised
ADOS: Autism Diagnostic Observation Schedule
ASD: Autism Spectrum Disorders
CARS-T: Childhood Autism Rating Scale-Translated
ELO: Evaluation of Oral Language
IQ: Intelligence Quotient
M: mean score
MCQ: Multiple choice questions
ODEDYS: Outil de Dépistage de la Dyslexie
RCPM: Raven's Colored Progressive Matrices board form
SD: Standard Deviation.
