# Supplementary figures and images for: Teaching Literacy Skills to French Minimally Verbal School-Aged Children with Autism Spectrum Disorders with the Serious Game SEMA-TIC: An Exploratory Study
Source: Front Psychol. 2017 Sep 5;8:1523. doi: 10.3389/fpsyg.2017.01523 (PMC5591836; doi:10.3389/fpsyg.2017.01523)

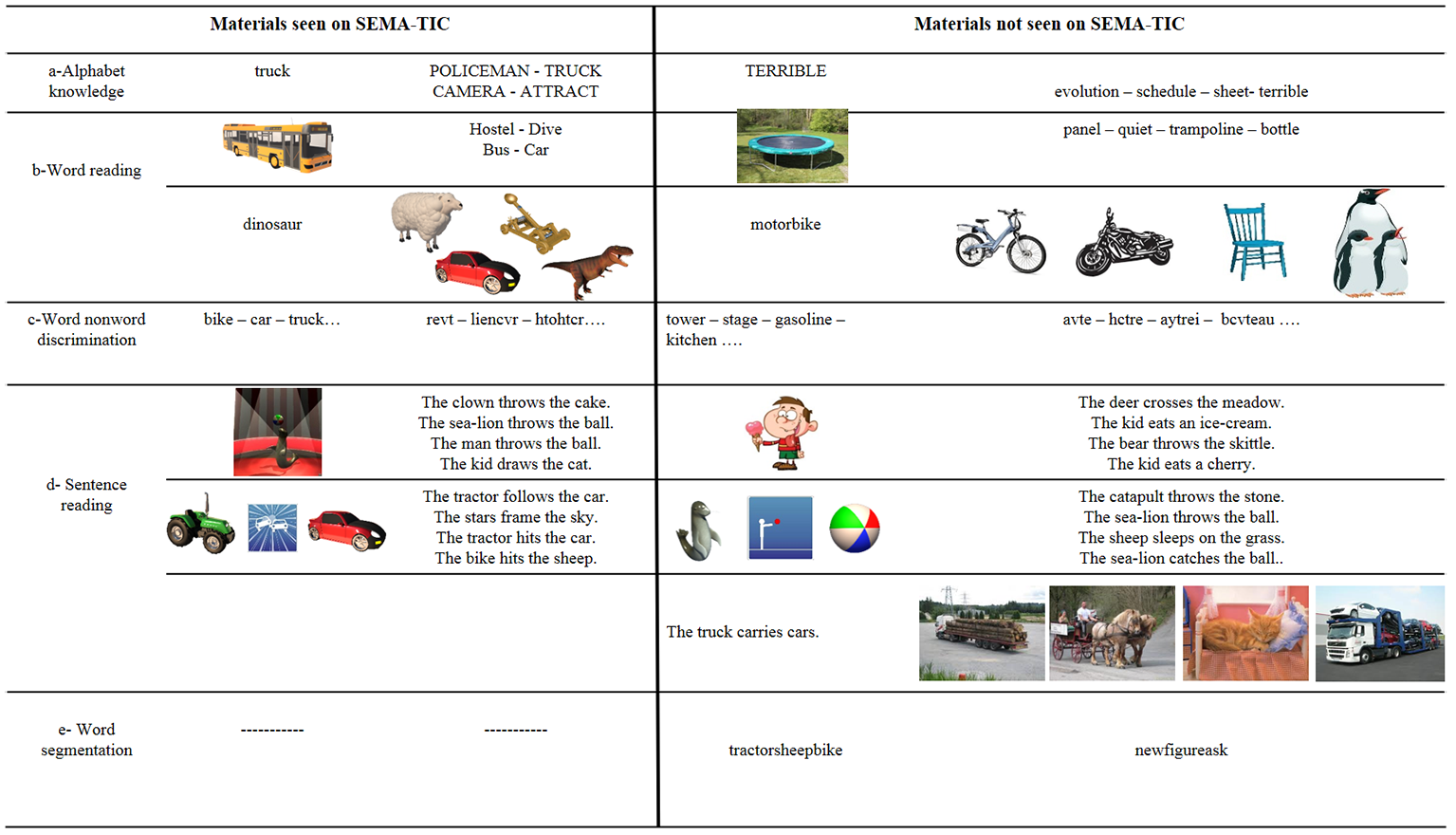

Supplement: Supplemental File 1 — Example of the items used in the experimental tasks. All materials have been translated to English for this paper. (A) Alphabet knowledge, (B) Word reading, (C) Word/non-word discrimination, (D) Sentence reading, (E) Word segmentation. [file SupplementalFile1.TIF]
