# Supplementary material for: Teaching Literacy Skills to French Minimally Verbal School-Aged Children with Autism Spectrum Disorders with the Serious Game SEMA-TIC: An Exploratory Study
Source: Front Psychol. 2017 Sep 5;8:1523. doi: 10.3389/fpsyg.2017.01523 (PMC5591836; doi:10.3389/fpsyg.2017.01523)
Supplement: Supplemental File 2 — Results of SEMA-TIC experimental tasks for typical developmental children (n = 16). [file SupplementalFile2.DOCX]

| **Children with typical development (n=16)** | **Mean** | **Standard deviation** | **Range** |
| --- | --- | --- | --- |
| **Alphabet knowledge** | 100 | 0 | 100-100 |
| **Word reading** | 99 | 2 | 93-100 |
| **Word nonword discrimination** | 91.6 | 5.8 | 80-100 |
| **Sentence reading** | 95.5 | 7.4 | 72-100 |
| **Word segmentation** | 79.6 | 22.3 | 28.7-100 |

Supplemental File 2: Results of SEMA-TIC experimental tasks for typical developmental children
