# Supplementary material for: Teaching Literacy Skills to French Minimally Verbal School-Aged Children with Autism Spectrum Disorders with the Serious Game SEMA-TIC: An Exploratory Study
Source: Front Psychol. 2017 Sep 5;8:1523. doi: 10.3389/fpsyg.2017.01523 (PMC5591836; doi:10.3389/fpsyg.2017.01523)
Supplement: Supplemental File 3 — Training and non-training groups' clinical and cognitive characteristics detailed at inclusion. [file SupplementalFile3.DOCX]

| **Training group** | **Age**  **(y,m)** | **Gender** | **Diagnosis**  **(DSM5)** | **Item 11**  **CARS-T** | **CARS-T** | **RCPM** | **Alouette**  **ODEDYS** | **Minimally verbal** | **ELO**  **Production of utterance**  **SD for 3 years** | **ELO**  **Repetion of utterance**  **SD for 3 years** |
| --- | --- | --- | --- | --- | --- | --- | --- | --- | --- | --- |
| 1 | 10,4 | m | ASD | 4 | 45 | 19 | 0 | No spoken language | -1.6 | -1.5 |
| 2 | 9,8 | m | ASD | 3.5 | 40 | 28 | 0 | Few spoken language | -1.6 | -1.5 |
| 3 | 7 | m | ASD | 3 | 34 | 16 | 0 | Few spoken language | -1.6 | -1.5 |
| 4 | 8,9 | m | ASD | 3 | 44 | 18 | 0 | Echolalic/stereotyped language | -0.6 | -1.5 |
| 5 | 8,6 | m | ASD | 3 | 34.5 | 19 | 0 | Few spoken language | -1.6 | -1.5 |
| 6 | 6,2 | f | ASD | 3.5 | 37.5 | 26 | 0 | Echolalic /stereotyped language | 0,1 | 0 |
| 7 | 6,5 | m | ASD | 3.5 | 44 | 18 | 0 | No spoken language | -1.6 | -1.5 |
| 8 | 8,3 | m | ASD | 3.5 | 34.5 | 18 | 0 | No spoken language | -1.6 | -1.5 |
| 9 | 11,4 | m | ASD | 3 | 43 | 17 | 0 | Echolalic/stereotyped language | 0.8 | -0.3 |
| 10 | 10,2 | m | ASD | 3.5 | 40 | 15 | 0 | Few spoken language | -0.9 | -1.5 |
| 11 | 6,8 | m | ASD | 3.5 | 38.5 | 26 | 0 | Echolalic/stereotyped language | -0.9 | -0,3 |
| 12 | 10,5 | m | ASD | 3.5 | 35.5 | 21 | 0 | Few spoken language | -1.6 | -1.5 |
| **Non-training group** | |  |  |  |  |  |  |  |  |  |
| 1 | 6,1 | m | ASD | 3 | 40 | 22 | 0 | Few spoken language | -1.6 | -1.5 |
| 2 | 9,4 | m | ASD | 3 | 39.5 | 25 | 0 | Few spoken language | -1.6 | -1.5 |
| 3 | 9,4 | m | ASD | 3 | 38.5 | 25 | 0 | Few spoken language | -1.6 | -1.5 |
| 4 | 7,11 | m | ASD | 3.5 | 39 | 19 | 0 | No spoken language | -1.6 | -1.5 |
| 5 | 10,3 | m | ASD | 3.5 | 38.5 | 15 | 0 | No spoken language | -1.6 | -1.5 |
| 6 | 8,7 | m | ASD | 3 | 38 | 23 | 0 | Echolalic/stereotyped language | 0.8 | 0.3 |
| 7 | 11,8 | m | ASD | 3.5 | 37 | 17 | 0 | Few spoken language | -1.6 | -1.5 |
| 8 | 6,5 | f | ASD | 4 | 43.5 | 18 | 0 | No spoken language | -1.6 | -1.5 |
| 9 | 6 | m | ASD | 3 | 31.5 | 18 | 0 | Echolalic/stereotyped language | -1.6 | -1.5 |
| 10 | 9,1 | f | ASD | 3 | 37.5 | 24 | 0 | No spoken language | -1.6 | -1.5 |
| 11 | 6,6 | m | ASD | 3 | 36 | 27 | 0 | Echolalic/stereotyped language | -0.9 | -1.5 |
| 12 | 8,11 | f | ASD | 3 | 34.5 | 18 | 0 | Echolalic/stereotyped language | -1.6 | -1.5 |
| 13 | 8,8 | f | ASD | 3 | 37.5 | 25 | 0 | Echolalic/stereotyped language | -0.2 | -1.5 |

Supplemental file 3: Training and non-training groups’ clinical and cognitive characteristics at inclusion
