# Supplementary material for: Teaching Literacy Skills to French Minimally Verbal School-Aged Children with Autism Spectrum Disorders with the Serious Game SEMA-TIC: An Exploratory Study
Source: Front Psychol. 2017 Sep 5;8:1523. doi: 10.3389/fpsyg.2017.01523 (PMC5591836; doi:10.3389/fpsyg.2017.01523)
Supplement: Supplemental File 4 — Individual performances in validated reading tests. [file SupplementalFile4.DOCX]

Supplemental file 4: Individual performances in validated reading tests

|  | **Alouette Reading Test** | | | | | | **ODEDYS** | | | | | |
| --- | --- | --- | --- | --- | --- | --- | --- | --- | --- | --- | --- | --- |
|  | **Pre-test** | | | **Post-test** | | | **Pre-test** | | | **Post-test** | | |
|  | **Number of letters read**  **( /5)** | **Number of isolated words read**  **(/ 10)** | **Number of words read in text**  **( /265)** | **Number of letters read**  **( /5)** | **Number of isolated words read**  **(/ 10)** | **Number of words read in text**  **( /265)** | **Regular words**  **( /20)** | **Irregular words**  **( /20)** | **Pseudo-words**  **( /20)** | **Regular words**  **( /20)** | **Irregular words**  **( /20)** | **Pseudo-words**  **( /20)** |
| **Training group** |  |  |  |  |  |  |  |  |  |  |  |  |
| Participant 1 | 0 | 0 | 0 | 0 | 0 | 0 | 0 | 0 | 0 | 0 | 0 | 0 |
| Participant 2 | 0 | 0 | 0 | 5 | 0 | 0 | 0 | 0 | 0 | 0 | 0 | 0 |
| Participant 3 | 0 | 0 | 0 | 5 | 10 | 35 | 0 | 0 | 0 | 15 | 14 | 15 |
| Participant 4 | 0 | 0 | 0 | 5 | 0 | 0 | 0 | 0 | 0 | 0 | 0 | 0 |
| Participant 5 | 0 | 0 | 0 | 5 | 0 | 0 | 0 | 0 | 0 | 0 | 5 | 0 |
| Participant 6 | 0 | 0 | 0 | 5 | 10 | 82 | 0 | 0 | 0 | 19 | 9 | 18 |
| Participant 7 | 0 | 0 | 0 | 0 | 0 | 0 | 0 | 0 | 0 | 0 | 0 | 0 |
| Participant 8 | 0 | 0 | 0 | 0 | 0 | 0 | 0 | 0 | 0 | 0 | 0 | 0 |
| Participant 9 | 0 | 0 | 0 | 5 | 10 | 39 | 0 | 0 | 0 | 8 | 2 | 11 |
| Participant 10 | 0 | 0 | 0 | 5 | 0 | 0 | 0 | 0 | 0 | 0 | 0 | 0 |
| Participant 11 | 0 | 0 | 0 | 5 | 0 | 0 | 0 | 0 | 0 | 0 | 0 | 0 |
| Participant 12 | 0 | 0 | 0 | 0 | 0 | 0 | 0 | 0 | 0 | 0 | 0 | 0 |
| **Non-training group** | | | | | | | | | | | | |
| Participant 1 | 0 | 0 | 0 | 0 | 0 | 0 | 0 | 0 | 0 | 0 | 0 | 0 |
| Participant 2 | 0 | 0 | 0 | 4 | 1 | 0 | 0 | 0 | 0 | 0 | 0 | 0 |
| Participant 3 | 0 | 0 | 0 | 5 | 3 | 0 | 0 | 0 | 0 | 0 | 0 | 0 |
| Participant 4 | 0 | 0 | 0 | 0 | 0 | 0 | 0 | 0 | 0 | 0 | 0 | 0 |
| Participant 5 | 0 | 0 | 0 | 0 | 0 | 0 | 0 | 0 | 0 | 0 | 0 | 0 |
| Participant 6 | 0 | 0 | 0 | 3 | 0 | 0 | 0 | 0 | 0 | 0 | 0 | 0 |
| Participant 7 | 0 | 0 | 0 | 5 | 0 | 0 | 0 | 0 | 0 | 0 | 0 | 0 |
| Participant 8 | 0 | 0 | 0 | 0 | 0 | 0 | 0 | 0 | 0 | 0 | 0 | 0 |
| Participant 9 | 0 | 0 | 0 | 0 | 0 | 0 | 0 | 0 | 0 | 0 | 0 | 0 |
| Participant 10 | 0 | 0 | 0 | 0 | 0 | 0 | 0 | 0 | 0 | 0 | 0 | 0 |
| Participant 11 | 0 | 0 | 0 | 5 | 0 | 0 | 0 | 0 | 0 | 0 | 0 | 0 |
| Participant 12 | 5 | 0 | 0 | 5 | 1 | 0 | 5 | 0 | 0 | 5 | 0 | 0 |
| Participant 13 | 0 | 0 | 0 | 5 | 0 | 0 | 0 | 0 | 0 | 0 | 0 | 0 |
