# Supplementary material for: Teaching Literacy Skills to French Minimally Verbal School-Aged Children with Autism Spectrum Disorders with the Serious Game SEMA-TIC: An Exploratory Study
Source: Front Psychol. 2017 Sep 5;8:1523. doi: 10.3389/fpsyg.2017.01523 (PMC5591836; doi:10.3389/fpsyg.2017.01523)
Supplement: Supplemental File 5 — Drop-outs' clinical and cognitive characteristics at inclusion, and performances at standardized and experimental tasks in pre-test. [file SupplementalFile5.DOCX]

| **Training group** | **Age**  **(y,m)** | **Gender** | **Diagnosis**  **(DSM5)** | **Item 11**  **CARS-T** | **CARS-T** | **RCPM** | **Alouette**  **ODEDYS** | **Minimally verbal** | **ELO**  **Production of utterance**  **SD for 3 years** | **ELO**  **Repetion of utterance**  **SD for 3 years** | **Raison drop out** |
| --- | --- | --- | --- | --- | --- | --- | --- | --- | --- | --- | --- |
| 1 | 9,2 | m | ASD | 3 | 41.5 | 18 | 0 | Echolalic/stereotyped language | 0.7 | 1.5 | Moved out |
| 2 | 6,2 | m | ASD | 3 | 38.5 | 15 | 0 | Few spoken language | 1.6 | -0.3 | Parents did not use SEMA-TIC at home |
| 3 | 8,0 | m | ASD | 3 | 37.5 | 15 | 0 | No spoken language | -1.6 | -1.5 | Parents did not use SEMA-TIC at home |
| **Non-training group** |  | | | | | | | | | | |
| 1 | 12,9 | m | ASD | 4 | 37.5 | 28 | 0 | No spoken language | -1.6 | -1.5 | Did not come at post-test evaluation |
| 2 | 6,0 | m | ASD | 3 | 33.5 | 15 | 0 | Few spoken language | 1.4 | 0.5 | Did not come at post-test evaluation |

Supplemental file 5: Drop-outs’ clinical and cognitive characteristics at inclusion, and performances at standardized and experimental tasks in pre-test

|  | **Standardized tests (pre-test)** | | | | | | **Experimental tasks (pre-test)** | | | | |
| --- | --- | --- | --- | --- | --- | --- | --- | --- | --- | --- | --- |
|  | **Alouette Reading Test** | | | **ODEDYS** | | | **AK** | **WR** | **WN**  **WD** | **SR** | **WS** |
|  | **Number of letters read**  **( /5)** | **Number of isolated words read**  **(/ 10)** | **Number of words read in text**  **( /265)** | **Regular words**  **( /20)** | **Irregular words**  **( /20)** | **Pseudo-words**  **( /20)** |  |  |  |  |  |
| **Training group** |  |  |  |  |  |  |  |  |  |  |  |
| Drop-out 1 | 0 | 0 | 0 | 0 | 0 | 0 | 40 | 10 | 15 | 0 | 2 |
| Drop-out 2 | 0 | 0 | 0 | 0 | 0 | 0 | 0 | 0 | 10 | 0 | 0 |
| Drop-out 3 | 0 | 0 | 0 | 0 | 0 | 0 | 0 | 3.3 | 15 | 0 | 0 |
| **Non training group** |  |  |  |  |  |  |  |  |  |  |  |
| Drop-out 4 | 0 | 0 | 0 | 0 | 0 | 0 | 30 | 25 | 20 | 0 | 0 |
| Drop-out 5 | 0 | 0 | 0 | 0 | 0 | 0 | 60 | 3.3 | 10 | 6.7 | 4 |
